# Alpha-Lipoic Acid Reduces Cell Growth, Inhibits Autophagy, and Counteracts Prostate Cancer Cell Migration and Invasion: Evidence from In Vitro Studies

**DOI:** 10.3390/ijms242317111

**Published:** 2023-12-04

**Authors:** Sabrina Bossio, Anna Perri, Raffaella Gallo, Anna De Bartolo, Vittoria Rago, Daniele La Russa, Michele Di Dio, Sandro La Vignera, Aldo E. Calogero, Giovanni Vitale, Antonio Aversa

**Affiliations:** 1 Department of Experimental and Clinical Medicine, University of Catanzaro “Magna Græcia”, 88100 Catanzaro, Italy; sabrina.bossio@unicz.it (S.B.); anna.perri@unicz.it (A.P.); 2 Laboratory of Immunology, Department of Experimental and Clinical Medicine, University of Catanzaro “Magna Græcia”, 88100 Catanzaro, Italy; r.gallo@unicz.it; 3 Cellular and Molecular Cardiovascular Pathophysiology Laboratory, Department of Biology, University of Calabria, 87036 Rende, Italy; anna.debartolo@unical.it; 4 Department of Pharmacy, Health and Nutritional Sciences, University of Calabria, 87036 Rende, Italy; vittoria.rago@unical.it; 5 Department of Biology, Ecology and Earth Sciences, University of Calabria, 87036 Rende, Italy; daniele.larussa@unical.it; 6 Division of Urology, Department of Surgery, Annunziata Hospital, 87100 Cosenza, Italy; micheledidio@yahoo.it; 7 Department of Clinical and Experimental Medicine, University of Catania, 95124 Catania, Italy; sandrolavignera@unict.it (S.L.V.); acaloger@unict.it (A.E.C.); 8 Department of Medical Biotechnology and Translational Medicine (BIOMETRA), University of Milan, 20133 Milan, Italy; giovanni.vitale@unimi.it; 9 Laboratory of Geriatric and Oncologic Neuroendocrinology Research, IRCCS Istituto Auxologico Italiano, 20145 Milan, Italy

**Keywords:** alpha-lipoic acid, prostate cancer cells, ROS, KEAP1, Nrf2, p62, autophagy, cell migration

## Abstract

Alpha-lipoic acid (ALA) is a natural antioxidant dithiol compound, exerting antiproliferative and antimetastatic effects in various cancer cell lines. In our study, we demonstrated that ALA reduces the cell growth of prostate cancer cells LNCaP and DU-145. Western blot results revealed that in both cancer cells, ALA, by upregulating pmTOR expression, reduced the protein content of two autophagy initiation markers, Beclin-1 and MAPLC3. Concomitantly, MTT assays showed that chloroquine (CQ) exposure, a well-known autophagy inhibitor, reduced cells’ viability. This was more evident for treatment using the combination ALA + CQ, suggesting that ALA can reduce cells’ viability by inhibiting autophagy. In addition, in DU-145 cells we observed that ALA affected the oxidative/redox balance system by deregulating the KEAP1/Nrf2/p62 signaling pathway. ALA decreased ROS production, SOD1 and GSTP1 protein expression, and significantly reduced the cytosolic and nuclear content of the transcription factor Nrf2, concomitantly downregulating p62, suggesting that ALA disrupted p62-Nrf2 feedback loop. Conversely, in LNCaP cells, ALA exposure upregulated both SOD1 and p62 protein expression, but did not affect the KEAP1/Nrf2/p62 signaling pathway. In addition, wound-healing, Western blot, and immunofluorescence assays evidenced that ALA significantly reduced the motility of LNCaP and DU-145 cells and downregulated the protein expression of TGFβ1 and vimentin and the deposition of fibronectin. Finally, a soft agar assay revealed that ALA decreased the colony formation of both the prostate cancer cells by affecting the anchorage independent growth. Collectively, our in vitro evidence demonstrated that in prostate cancer cells, ALA reduces cell growth and counteracts both migration and invasion. Further studies are needed in order to achieve a better understanding of the underlined molecular mechanisms.

## 1. Introduction

Prostate cancer is among the most common solid malignancies, the prognoses of which vary widely according to age, ethnicity, genetic background, and, above all, tumor grade and stage at primary diagnosis. Thus, the life expectancy for men with localized and early-stage prostate cancer can be as high as 99% over 10 years, while men who are diagnosed with late-stage disease have an overall survival at 5 years of 30% [1]. The androgen receptor (AR) is one of the most studied and therapeutically targeted oncogenes in prostate cancer, but androgen deprivation therapy (ADT), as well as AR signaling inhibitors, frequently lead to alterations of AR expression or post-translational modifications, resulting in resistance to therapy over time, occurring via multiple mechanisms that are still undergoing investigation [2]. Despite the availability of multiple classes of therapy, metastatic castration-resistant prostate cancer is associated with a poor prognosis and worse overall survival rates. Therefore, the future challenge is classifying prostate cancer into high and low risk of disease progression under ADT and developing therapeutic strategies based on personalized genetic and cellular profiles combined with standard risk factors and therapies.

Also known as “self-eating”, autophagy is a catabolic process playing an essential role in cellular homeostasis, since it promotes the generation of energy required for cellular equilibrium, especially during nutrient deprivation. In cancer cells, autophagy shows both protective and detrimental effects. On the one hand, it can support cell growth by facilitating nutrient recycling, mainly upon hypoxia conditions; on the other hand, the excessive degradation of cellular components can cause cancer cell death [3]. Interestingly, autophagy can regulate the proliferation and metastasis capacity of prostate cancer cells and its manipulation can affect how prostate cancer cells respond to chemotherapy and radiotherapy, potentially influencing the therapeutic outcome [4,5,6,7].

In recent decades, several researchers have demonstrated that some nutraceuticals can counteract tumor development and progression by targeting the cancerous cells at multiple levels, such as by promoting cell cycle arrest or apoptosis, suppressing metastasis, invasion, angiogenesis, and by modifying the redox status and the tumor microenvironment Furthermore, some natural bioactive compounds are able to resensitize drug-resistant tumors via their pleiotropic capability to affect different intra-cellular pathways [8]. Interestingly, a recent extensive review by Hitesh Chopra et al. described the main experimental evidence demonstrating the effectiveness of nanonutraceutical approaches in the management of prostate cancer, highlighting that nutraceuticals could represent a promising therapeutic tool in prostate cancer therapy [9].

A growing number of studies have demonstrated that α-lipoic acid (ALA), a natural dithiol compound with marked antioxidant properties, displays antiproliferative and antimetastatic potential in various cancer cell lines by affecting different oncogenic signaling pathways. Furthermore, favorable results emerging from a few preclinical cancer models and human studies with ALA and its derivatives have opened promising scenarios for their potential application either alone or in combination with anticancer drugs in the management of tumors of different origins [10,11,12,13,14,15,16,17,18,19,20,21,22,23,24,25,26].

In this study, prompted by these emerging findings, we have investigated the effects of ALA exposure in both AR^+^ and AR^−^ prostate cancer cell lines, LNCaP and DU-145, respectively.

## 2. Results

### 2.1. ALA Reduces Prostate Cancer Cells’ Viability by Inhibiting Autophagy

**Figure 1:**
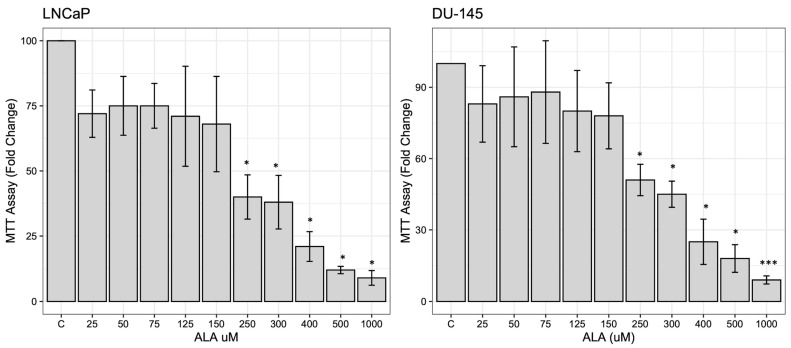
MTT assays in LNCaP and DU-145 cells were either treated or not with increasing doses (25–1000 μM) of ALA for 48 h. Cell proliferation is expressed by fold change ± standard deviations (SD), with respect to basal conditions, and is representative of three independent experiments, each performed in eight replicates. Statistical significance was considered at * *p* < 0.05; *** *p* < 0.0001. Statistical comparisons were drawn between groups using a two-tailed *t*-test (C) = control.

A cell viability assay was used to investigate the cytotoxic effects of ALA exposure in prostate cancer cell lines, LNCaP and DU-145. To this purpose, the cells were synchronized in SFM for 12 h and then treated with increasing doses of ALA (25–1000 μM) for 48 h. MTT assay results demonstrated that ALA exposure promoted a significant dose-dependent reduction in cell viability (Figure 1a), with a half-maximal inhibitory concentration (IC_50_), estimated as 271 μM for LNCaP (*p* = 0.028) and 278 μM for DU-145 (*p* = 0.020). Therefore, these concentrations were used for all the successive experiments.

**Figure 2:**
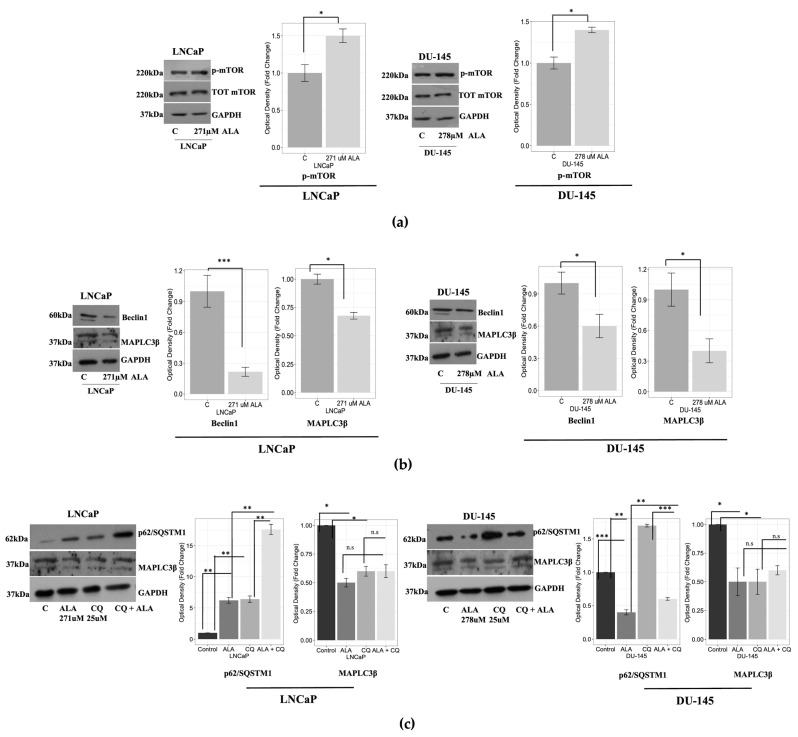

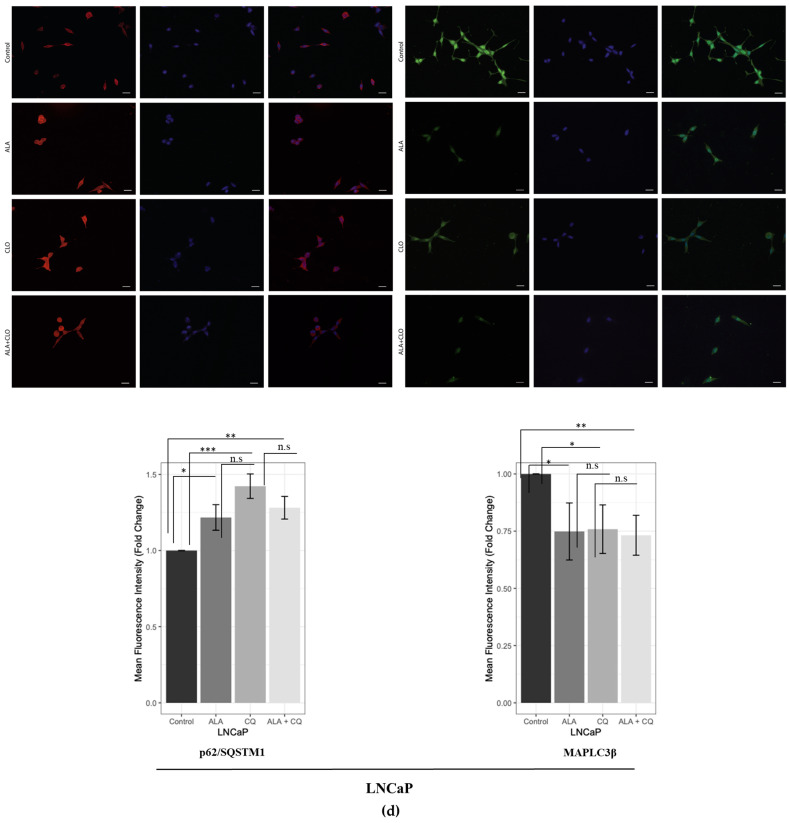

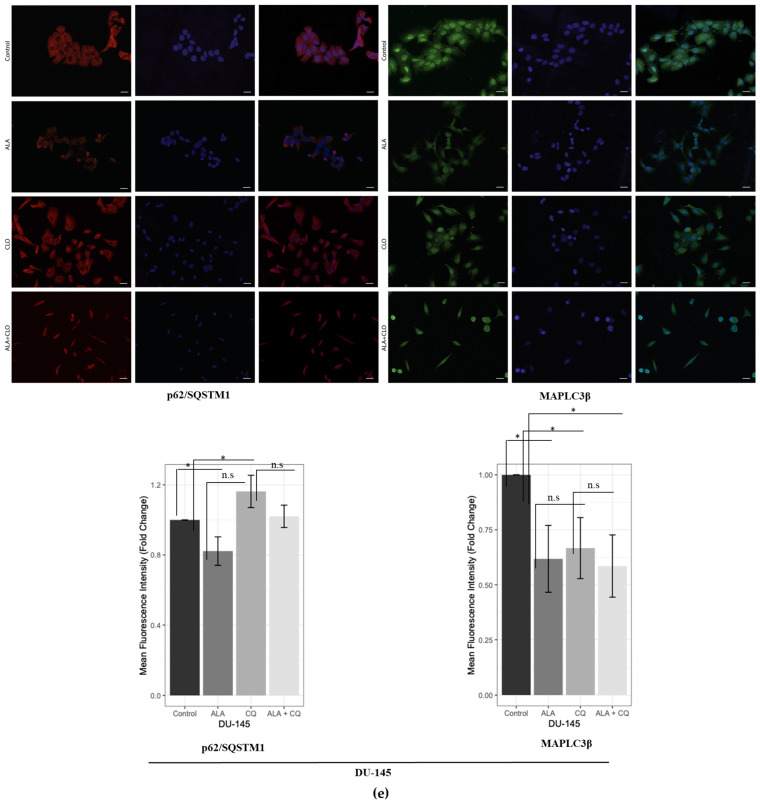

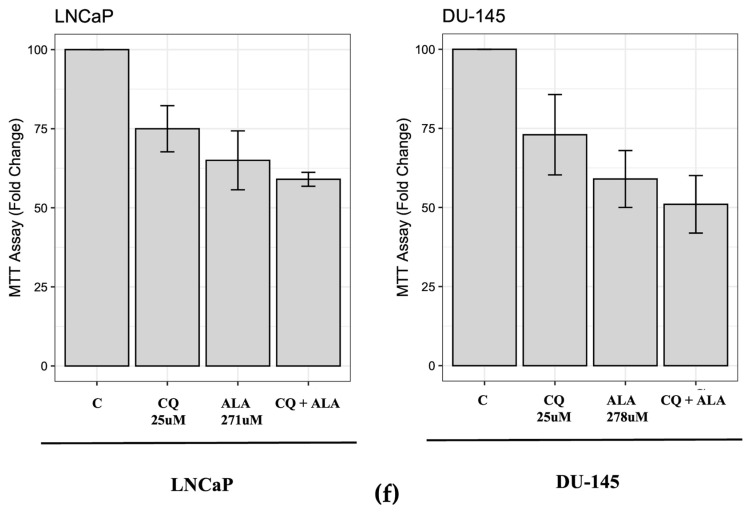
(**a**) Immunoblotting analysis showing an increased protein expression of p-mTOR in both cell lines after exposure for 48 h to ALA. GAPDH was used as a loading control. The bars represent the mean ± SD of three experiments in which the band intensities were evaluated as the optical density and are represented as fold change for treated vs. untreated cells normalized for the loading control. * *p* < 0.05 treated vs. untreated cells. (**b**) Western blotting analysis showing protein reduction in Beclin-1 and MAPLC3β protein expressions in LNCaP and DU-145 cells. GAPDH was used as a loading control. The bars represent the mean ± SD of three experiments in which band intensities were evaluated as the optical density and are represented as fold change for treated vs. untreated cells normalized for the loading control. * *p* < 0.05; *** *p* < 0.0001 treated vs. untreated cells. (**c**) Immunoblotting analysis showing protein expression of SQSMT1/p62 and MAPLC3β after treatment with ALA and CQ alone and in combination (CQ + ALA). The optical density is evaluated as fold change for treated vs. untreated cells normalized for the loading control (** *p* <0.001; *** *p* < 0.0001) and ALA and CQ treatment vs. CQ + ALA exposure normalized for the loading control. (* *p* < 0.05; ** *p* < 0.001; *** *p* < 0.0001); n.s not significant. (**d**,**e**) IF of SQSMT1/p62 and MAPLC3β expression in LNCaP cells (**d**) and DU-145 (**e**) treated with ALA, CQ and CQ + ALA. The bars represent the mean ± SD fluorescence intensity, represented as fold change for treated vs. untreated cells and ALA and CQ treatment vs. CQ + ALA * *p* < 0.05; ** *p* < 0.001; *** *p* < 0.0001. Scale bar: 25 μm (**f**) MTT assay in prostate cancer cells. LNCaP and DU-145 cells were treated or not with 25 μM of CQ, ALA and in combination CQ + ALA. Cell proliferation is expressed as fold change ± standard deviations (SD), with respect to basal conditions, and is representative of three independent experiments, each performed in eight replicates. Statistical significance was considered at * *p* < 0.05; ** *p* < 0.001; *** *p* < 0.0001. Statistical comparisons were drawn between groups using a two-tailed *t*-test (C) = control.

To establish whether the reduced viability induced by ALA and observed in LNCaP and DU-145 cells could be attributed to autophagy inhibition, we first investigated the expression of p-mTOR, since in vitro evidence reported that ALA can inhibit the autophagic flux via the activation of its negative regulator, p-mTOR [27]. Next, we evaluated the protein expression level of key molecular markers of the autophagy machinery initiation. Interestingly, Western blot results revealed that, in both cell lines, ALA significantly upregulated p-mTOR protein expression, concomitantly with the downregulation of Beclin-1 and MAPLC3β (Figure 2a,b). In order to confirm the capability of ALA to reduce autophagic flux, we performed Western blot (Figure 2c) and IF (Figure 2d,e) assays, treating cells with a wide autophagy inhibitor, chloroquine (CQ) (25 μM), and ALA alone or in combination with CQ. As expected, CQ promoted p62/SQTM1 accumulation and decreased MAPLC3β expression in both cancer cells (Figure 2c–e). Similar results were observed in LNCaP and DU-145 cells exposed to ALA, except for p62/SQTM1, which was downregulated upon ALA exposure in DU-145 cells (Figure 2c–e). Finally, no significant difference was observed for MAPLC3β in both cancer cell lines exposed to combined treatment CQ + ALA vs. CQ or ALA alone (Figure 2c,d). Conversely, we observed a significant modulation of p62/SQTM1 in LNCaP and DU-145 cells exposed to the combined treatment CQ + ALA vs. ALA and CQ alone (Figure 2c–e).

Next, to strengthen the hypothesis that autophagy inhibition can impair cancer cells’ viability, we performed the MTT assay in cells exposed to CQ, ALA, and ALA + CQ. The MTT assay showed that, in both cancer cell lines, CQ significantly reduced cell viability (*p* < 0.03 for LNCaP; *p* < 0.002 for DU-145) and that the combined treatment CQ + ALA promoted a significant cell viability reduction compared to CQ and ALA alone (*p* < 0.001 for LNCaP; *p* < 0.001 for DU-145) (Figure 2f). This data suggests that ALA could affect prostate cancer cells’ viability by inhibiting autophagy and that the compound could exert an additive antiproliferative effect when combined with an autophagy inhibitor.

### 2.2. ALA Affects the Oxidative/Redox System Balance of Prostate Cancer Cells

**Figure 3:**
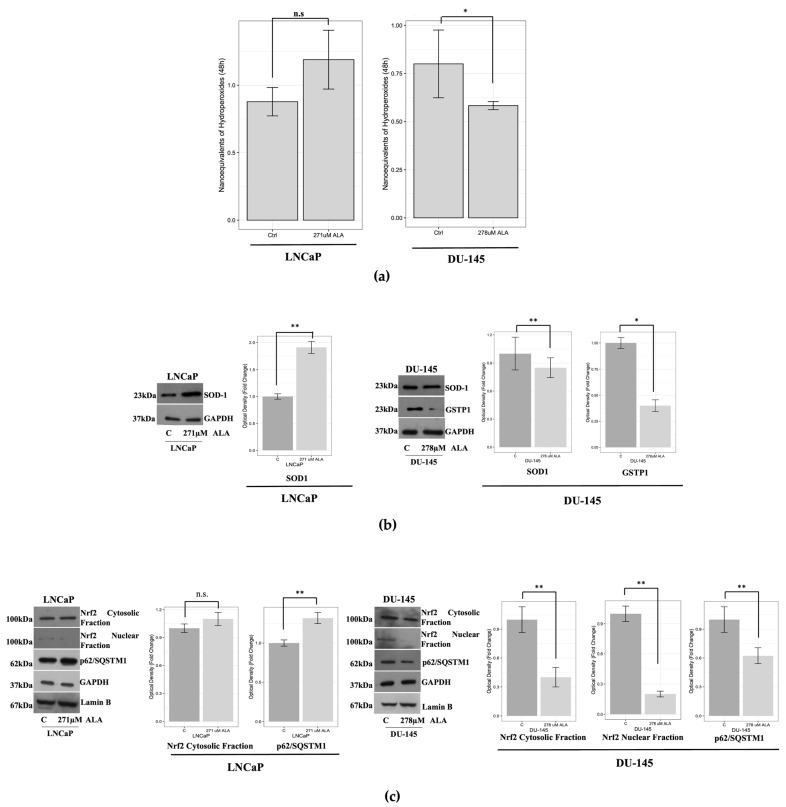
(**a**) Nanoequivalents of hydroperoxides detected by Lipocell assay in untreated (control) and treated cells. n.s. not significant; * *p* < 0.05 (**b**) Immunoblotting showing SOD-1 and GSTP1 protein expressions in LNCaP and DU-145 cells after treatment for 48 h with ALA. For LNCaP GSTP1, protein expression is undetectable, as described in the text below. GAPDH was used as a loading control. The bars represent the mean ± SD of three experiments. Optical density was represented as fold change for treated vs. untreated cells normalized for the loading control. * *p* < 0.05; ** *p* < 0.001 treated vs. untreated cells. (**c**) Western blotting of the cytosolic and nuclear protein extracts shows that in LNCaP there was a low increase in cytosolic fraction, depending on whether the nuclear fraction was undetectable after exposure to ALA. Instead, in DU-145 cells, a significant reduction in cytosolic and nuclear fraction of Nrf2 protein expression after exposure to ALA was observed. GAPDH and Lamin B were used as loading controls. Immunoblotting of p62/SQSTM1 showed an upregulation of protein expression in LNCaP cells and a downregulation in the DU-145 cell line. The bars represent the mean ± SD of three experiments in which the band intensities were evaluated as the optical density and are represented as fold change for treated vs. untreated cells normalized for the loading control. ** *p* < 0.001 treated vs. untreated cells; n.s. not significant.

Various in vitro cancer models have demonstrated that ALA counteracts cancer progression by affecting the oxidative/redox system balance of cancer cells [28]. Therefore, in our experiment models, we investigated whether ALA exposure modulates the ROS content in both cell lines. Interestingly, in LNCaP cells, we observed an almost significant increase in ROS content (*p* = 0.06) after ALA compared to the control, concomitantly with a significant upregulation of the antioxidant enzyme SOD1 after 48 h. Conversely, in the same experimental conditions, our results revealed that in DU-145 cells, ALA exposure significantly reduced ROS production (*p* < 0.001) as well as SOD-1 and GSTP1 protein expression levels (Figure 3a,b).

Conversely, the protein expression of GSTP1 in LNCaP cells was not detectable, since it is well known that the hypermethylation of the CpG islands at the promoter of GSTP1 that occurs during prostate carcinogenesis leads to transcriptional inactivation [29].

Next, we explored whether ALA was able to modulate the activation of the transcription factor Nrf2, playing an important role in response to oxidative stress and promoting antiapoptotic and prosurvival actions in cancer cells [30]. Our Western blot results demonstrated that, in LNCaP cells, Nrf2 was undetectable in the nuclear compartment and that ALA treatment did not modulate its cytosolic content (Figure 3c). Interestingly, in DU-145 cells, Nrf2 was also expressed in the nuclear compartments of untreated cells, suggesting its basal overactivation and that ALA significantly decreased its content in both cytosolic and nuclear compartments (Figure 3c). It has been reported that p62/SQSTM1 competes with Nrf2 to bind to KEAP1, promoting the dissociation of NRF2-KEAP1 and activating NRF2, which can in turn directly induce p62 transcription, thus forming a p62-NRF2 feedback loop [31]. This evidence could explain the downregulation of p62/SQSTM1 observed upon ALA exposure despite the fact that the compound inhibits autophagy (Figure 2c and Figure 3c). Conversely, in LNCaP cells, ALA increased p62/SQSTM1 expression (Figure 3c), most likely because in this prostate cancer cell line ALA does not affect the Nrf2/KEAP1/p62/SQSTM1 axis or the oxidative/redox balance system.

### 2.3. ALA Counteracts Prostate Cancer Cell Motility by Reducing Epithelial to Mesenchymal Transition (EMT)

**Figure 4:**
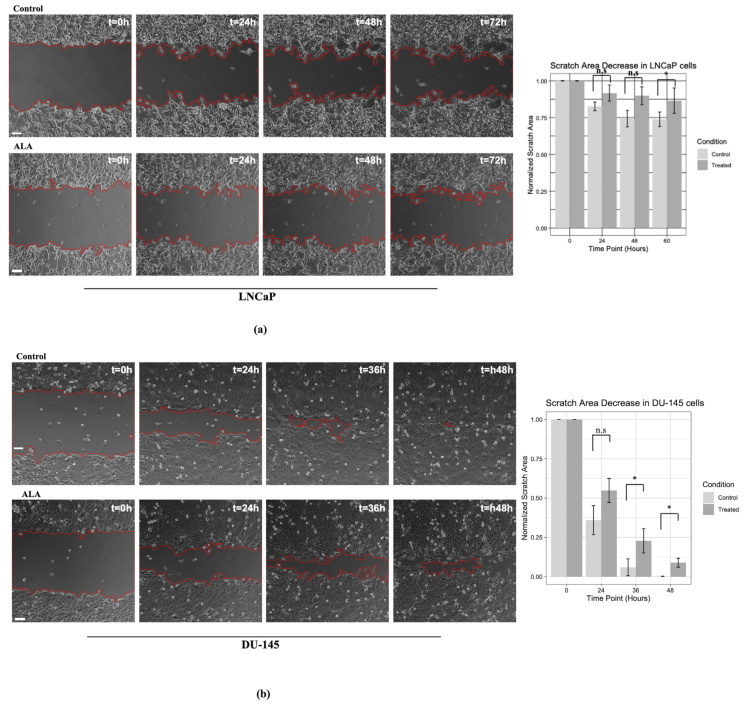
A wound-healing assay was performed as described previously. In (**a**) we showed the scratch in LNCaP cell lines (untreated vs. ALA) and in (**b**) the DU-145 cells (control vs. ALA). Cell migration was monitored by using a Leica DMi8 inverted microscope (Leica THUNDER Imager Live Cell, Wetzlar, Germany). Time-lapse images were captured every 20 min for 72 h, using an ×10 objective and a phase contrast filter. The bars showed a normalized area of each cell line after ALA treatment with respect to the untreated cell. The ImageJ software (version 2.1.0/1.53c) was used to analyze the time-lapse images as previously described [32] with the following parameters: variance window radius 20, threshold value 20, percentage of saturated pixels 0.001 and variance window radius 20, threshold value 30, percentage of saturated pixels 0.001 for DU-145 and LNCaP cells, respectively. Statistics and figures were obtained using R (R version 4.2.1, RStudio version 2021.09.0). Scale bar: 100 μm, * *p* < 0.05 treated vs. untreated cells; n.s. not significant.

We assessed a wound-healing assay in order to investigate the ability of ALA to counteract the migratory properties of prostate cancer cell lines. Our results revealed that ALA treatment significantly counteracted wound closure (Figure 4a) compared with the untreated cells. Interestingly, we observed that the antimigratory effects promoted by ALA treatment were more evident in DU-145 cells than in LNCaP cells. Data collection showed that in DU-145 cells, there was a wound closure at 24 h (*p* = 0.054) that resulted in significant reduction after 48 h of treatment (*p* = 0.033). Instead, in treated LNCaP cells, we observed decreased cell migration with a less marked wound closure over time, but with a significant reduction after 72 h of treatment (*p* = 0.042) (Figure 4a,b).

The images provided in Supplementary Video S1 documented that ALA reduces the migratory capacity of both cancer cell lines, although a different rate of migration resulted between LNCaP and DU-145 cells. These findings were further strengthened by the Western blot results, demonstrating that ALA counteracted cancer cell migration by affecting players driving the epithelial–mesenchymal transition (EMT) process.

**Figure 5:**
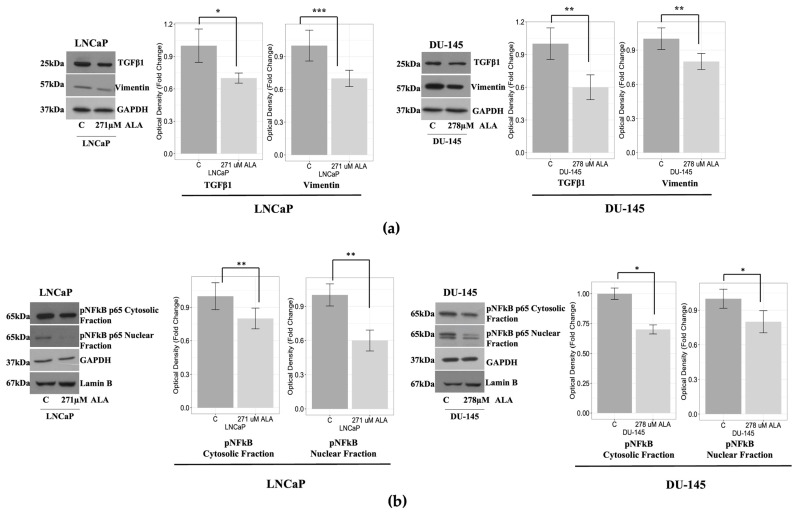

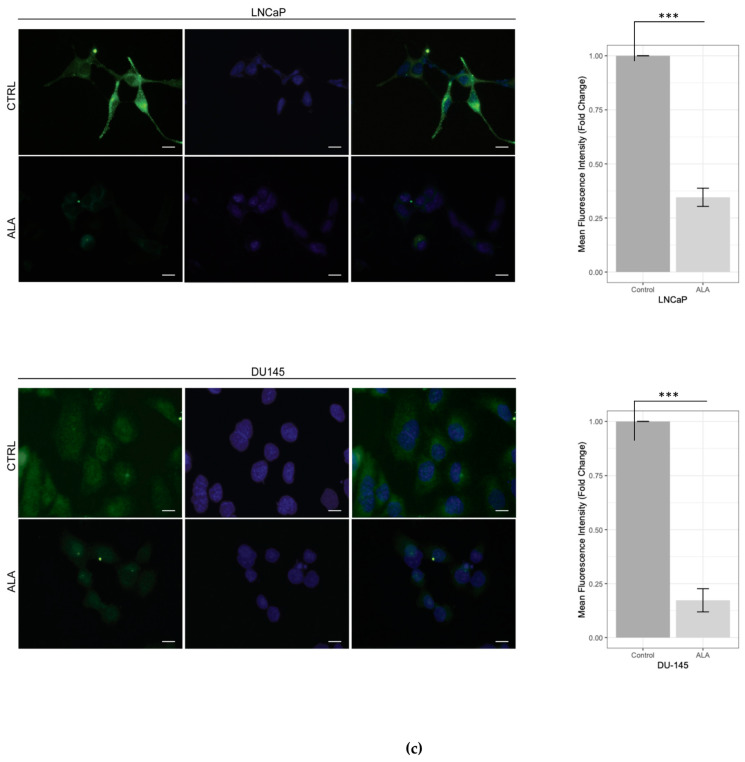
(**a**) Western blotting analysis shows a downregulation of TGFβ-1 and Vimentin proteins expression in both LNCaP and DU-145 after ALA treatment for 48 h. The bars represent the mean ± SD of three experiments. Optical density was evaluated as the optical density and is represented as fold change for treated vs. untreated cells normalized for the loading control. * *p* < 0.05; ** *p* < 0.001; *** *p* < 0.0001 treated vs. untreated cells. GAPDH was used as loading control. (**b**) Immunoblotting showing pNF-kB protein expressions in LNCaP and DU-145 cells exposed for 48 h to ALA. GAPDH was used as a loading control. The bars represent the mean ± SD of three experiments in which band intensities were evaluated as the optical density and are represented as fold change for treated vs. untreated cells normalized for the loading control. * *p* < 0.05; ** *p* < 0.001 treated vs. untreated cells. (**c**) Immunofluorescence assay (IF) of fibronectin protein was assessed for both prostate cancer cell lines. Cell monolayers were seeded and then treated with ALA for 24 h. The image showed a reduction in the signals of the fibronectin after ALA treatment (third panels) in both cell lines compared to untreated cells. The bars represent the mean ± SD of three experiments. Mean fluorescence intensity is represented as fold change for treated vs. untreated cells. *** *p* < 0.0001. Scale bar: 12.5 μm.

Interestingly, we observed that ALA promoted a significant downregulation of TGFβ1 and Vimentin in both cell lines (Figure 5a), strongly suggesting that ALA could counteract the EMT process. In addition, we evaluated the expression of the transcription factor NF-kB, promoting survival, proliferation, and invasion in prostate cancer [33,34], and whose activation is reduced by ALA in other cancer cell models [25]. By using cytosolic and nuclear fractions of both cell lines, we observed that, in LNCaP and DU-145 cells, ALA significantly reduced the cytosolic and nuclear content of NF-kB, suggesting that its inhibition could be involved in the antimigratory and antiproliferative effects promoted by ALA (Figure 5b).

This data was also confirmed by immunofluorescence assay (IF), which was performed in both cell lines treated for 24 h with ALA (Figure 5c). The fluorescent microscopy image documented that fibronectin’s expression was downregulated after ALA treatment, further confirming that ALA efficaciously counteracts the EMT process.

### 2.4. ALA Inhibits the Colony-Forming Ability of Prostate Cancer Cell Lines

**Figure 6:**
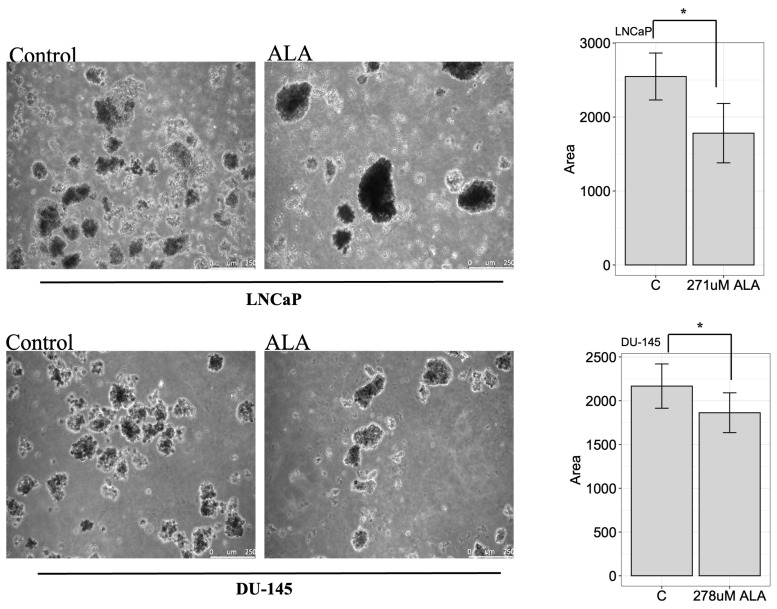
A soft agar assay was conducted on LNCaP and DU-145 untreated and treated with ALA. The images were taken after 15 days using a Leica DMi8 inverted microscope. The figure shows untreated cells on the left, with a mean area of colony of 2.548 ± 317.2 (mean ± SD) for LNCaP cells and 2.167 ± 252.9 for DU-145 cell lines (**left panels**). Meanwhile, the treated cells (**right panels**) showed a significance reduction in colony area of 1.782 ± 401.3 and 1.862.8 ± 227.3 for LNCaP and DU-145, respectively. Colony formation was reduced in both cell lines in the presence of treatment. The histograms show a reduction in colony area both in LNCaP and in DU-145 cell lines. For statistical analysis we used the ImageJ software (version 2.1.0/1.53c). * *p* < 0.05 treated vs. untreated cells. Scale bar: 250 μm.

Finally, to imitate in vivo the cancer cell growth, we performed a soft agar assay. LNCaP and DU-145 were seeded as described previously and were treated with ALA. After two weeks, colonies were photographed using a Leica DMi8 inverted microscope (Leica THUNDER Imager Live Cell) and we observed that ALA significantly inhibited the colony forming area to 1.782 ± 401.2 (*p* < 0.05) and 1862.8 ± 227.3 (*p* < 0.05) in LNCaP and DU-145, respectively, compared to untreated cells (2.548 ± 317.2, LNCaP; 2167.2± 252.9, DU-145) (Figure 6). Interestingly, our data suggested that ALA could play an important role in the tumorigenic mechanism.

## 3. Discussion

In this in vitro study, we have demonstrated that in AR^+^ and AR^−^ prostate cancer cells, α-lipoic acid (ALA) reduces cell proliferation by inhibiting autophagy, and efficaciously counteracts cellular migration and invasion.

ALA is a disulfide compound synthesized from octanoic acid in the mitochondria; it may also be found in the human diet, such as in meat, potatoes, and fresh egg yolk. ALA is well known for its powerful antioxidative effects and is commonly used in the treatment of chronic inflammatory diseases associated with high levels of oxidative stress, such as diabetic polyneuropathy, Alzheimer’s disease, and renal diseases [35,36,37]. A growing body of evidence has demonstrated that ALA can also suppress the growth, migration, and invasion of various cancer cell lines, although the underlying mechanisms of action of ALA are complex and vary according to the type of cancer cell [10,11,12,13,16,17,18,19,20,21,22,23,24,25,26,27,38,39]. Few studies have investigated the cytotoxic effect of ALA in human prostate cancer cells, demonstrating that the compound, alone or in combination with other natural bioactives, promotes cell apoptosis [14,15]. Our results suggest that the reduced cell viability induced by ALA and observed in both cancer cell lines, could occur via mTOR-mediated inhibition of autophagy. In both cell lines, we found that ALA significantly reduced the expression of two key masters of autophagy initiation: Beclin-1 and LC3/I/II. We further confirm the prosurvival effect of autophagy machinery in prostate cancer cells, since treatment with chloroquine reduced cellular viability in both cell lines and cotreatment with ALA promoted further reduction in viability. The key role of autophagy in prostate cancer progression and androgen and chemoresistance processes has been discussed recently by Loizzo D et al., highlighting that the genetic signature of autophagy could be a useful tool to stratify prostate cancer aggressiveness [6]. Interestingly, the overexpression of the major autophagy proteins, such as Beclin-1, LC3I/II, and p62, is associated with a high Gleason score and extraprostatic invasion [40]. The results emerging from preclinical models, showing that the inhibition of autophagy promotes cell death, have been shifted into clinical trials involving patients with refractory prostate cancer; however, the available evidence still lacks clinical feasibility and effectiveness, most likely because of the complexity of both prostate cancer biology and autophagy machinery functioning [41,42]. The effect promoted by ALA treatment through autophagy inhibition has also been demonstrated in lung and breast cancer cells [17,27]. Interestingly, Chakravarti B et al. described autophagic flux blockade resulting from ALA-induced ROS generation in breast cancer cells. It is well known that in cancer there is an imbalance in the oxidant/reduction reactions and that the pro-oxidant environment surrounding cancer cells offers them many survival advantages, favoring tumor progression [17,43]. In prostate cancer, antiandrogen therapy causes hormone deprivation-induced oxidative stress, ROS production, and amplified redox signaling networks, facilitating the selection of castrate-resistant prostate cancer cells that can metastasize to distant sites [44]. Furthermore, ROS can either activate or prevent autophagy, depending on the cellular microenvironment and, in turn, autophagy can eliminate the sources of ROS production or, conversely, can promote ROS production [45]. In this scenario, a key role is played by the KEAP1/NrF2/p62 signaling pathway, the main regulator of antioxidant enzymes. The persistent Nrf2 activation and the adaptive antioxidant response observed in many cancers protects cells from the oxidative damage caused by chemotherapy and radiotherapy, concomitantly promoting cell survival and proliferation [46]. Our findings showed that in AR^−^ prostate cancer cells, DU-145, ALA treatment significantly reduced ROS production, simultaneously downregulating the protein expression of two key antioxidant enzymes, SOD1 and GSTP1, thus suggesting that ALA may affect the oxidative/redox imbalance of prostate cancer cells that favor and sustain cancer cells survival. Furthermore, in the same experimental conditions, we observed that ALA reduced the nuclear translocation of Nrf2. Canonical activation of Nrf2 occurs when KEAP1 is oxidized, triggering its dissociation and allowing stabilized Nrf2 to translocate into the nucleus and activate the expression of numerous antioxidant, detoxification, antiapoptotic, and prosurvival genes [30]. p62, a marker of autophagy degradation, competes with NRF2 to bind KEAP1 through the KIR domain, promoting the dissociation of NRF2-KEAP1 and activating NRF2 [31]. In addition, Nrf2 can directly induce p62 transcription and its protein expression after activation, thus forming a p62-NRF2 feedback loop [46,47]. In line with the above-reported literature data, our results showed that in DU-145 cells, concomitantly with decreased Nrf2 nuclear content, ALA decreased p62 protein expression, indicating that the biological effect induced by ALA and observed in DU-145 cells may also be a consequence of p62-NRF2 feedback loop disruption. Moreover, this mechanism could explain the evidence that, despite the capability of ALA to inhibit the autophagic flux, in DU-145 cells it promotes p62/SQTM1 downregulation. The relevance of the p62/Keap1/Nrf2 axis in cancer cells has been well described recently by Wei-Lun Hsu et al., who have reported that, in colon cancer cells exhibiting persistent Nrf2 activation, a natural compound interfered in the positive feedback loop p62-Keap1-Nrf2 axis of the noncanonical Keap1-Nrf2 pathway, concomitantly inhibiting autophagy activation and resulting in the death of cancer cells [47,48]. Overall, these findings fit well with the data reported in the literature, highlighting that natural compounds alone or in combination with androgen deprivation therapy or radiotherapy could have a relevant clinical impact on prostate cancer care, by changing Nrf2 status and prostate cancer cells’ redox homeostasis [49].

A further interesting effect exhibited by ALA in our cellular models is its ability to efficaciously counteract the migratory potential of prostate cancer cells, which we addressed to the downregulation of the key master driving the epithelial–mesenchymal transition process, TGFβ1, which was observed in both cell lines treated with ALA. Furthermore, the mitigation of EMT by ALA is further demonstrated by the significant downregulation of the mesenchymal marker, vimentin, and by the reduced expression of the main components of the extracellular matrix secreted by myofibroblast, fibronectin [50]. Our findings agree with previous studies performed in different cultured tumor cell models, providing evidence that ALA blocks invasion and metastasis by repressing TGFβ-induced EMT and the activity of matrix metalloproteinases markers [11,21,51].

Overall, our in vitro results have demonstrated that ALA can affect two cellular pathways playing a significant role in the progression of prostate carcinoma: Nrf2/Keap1/p62 and autophagy machinery, as recently highlighted in the literature [6,49]. Further studies are needed to better explain the molecular mechanisms underlying the anticancer effects promoted by ALA in both AR^+^ and AR^−^ prostate cancer cells. Furthermore, despite all experimental studies and multiple clinical trials demonstrating the health benefits of ALA, such as its antitumor effects, its clinical use is still widely discussed because of its short half life and low bioavailability (30%). In addition, although the nanoformulations increase ALA bioavailability, ALA can change the metabolism of co-administered drugs which are taken in combination. Finally, LC-MS/MS chromatography has demonstrated that the degradation of ALA and its related analogs by gut microbiota leads to the generation of metabolic products that can be toxic for various organs [52]. The safe dose for action of ALA was reported to be between 300 and 1800 mg per day [52]. In our in vitro models we used ~60 µg/mL of ALA; therefore, the potential anticancer effect in vivo could be achieved by using ALA doses not exceeding those considered as safe. However, considering the above-reported limitations, further studies are need in order to consider ALA as a promising new therapeutic tool for the treatment of prostate carcinoma.

## 4. Materials and Methods

### 4.1. Cell Culture and Treatment

The prostate cancer cell lines LNCaP and DU-145 were cultured in an RPMI-1640 culture medium, supplemented with 10% fetal bovine serum, 1% glutamine, and 1 mg/mL penicillin/streptomycin (Sigma Aldrich, Milan, Italy). Cells were cultured in 100 mm dishes and were kept incubated at 37 °C in an atmosphere of 5% CO_2_. All experiments were performed after 12 h of cell synchronization in serum-free media (SFM). The cells were exposed to the following treatments: alpha-lipoic acid (ALA) (Uriach Italy SrL, Asiago (Milan), Italy), which was dissolved in sterile dimethyl sulfoxide (DMSO, Sigma Aldrich, Milan, Italy) and diluted in RPMI-1640 media before use; chloroquine (25 µM; Sigma Aldrich, Milan, Italy, C6628).

### 4.2. Cell Viability Assay

Cell viability was determined using the 3-(4,5-dime-thylthiazol-2-yl)-2,5-diphenyltetrazolium (MTT) assay. Briefly, the cells were implanted in 96-well plates at a density of 4 × 10^4^ for LNCaP and 3 × 10^4^ for DU-145 and were synchronized in SFM for 12 h. The cells were exposed to treatments with increasing doses of ALA (25–1000 μM) for 48 h, as this experimental time equalized the cell division length of both types of prostate cells [53]. Eight replicates were performed for each sample. Then, 20 μL of MTT (5 mg/mL) was added to the cell media, and after 4 h of incubation, isopropanol (200 μL) was added to each well and the optical density was measured at 570 nm using a Beckman Coulter microplate reader. For the calculation of IC_50_, the tool’s calculation of AAT Bioquest was used (AAT Bioquest, Inc. Pleasanton, CA, USA (12 June 2023). Quest Graph™ IC50 Calculator. AAT Bioquest. https://www.aatbio.com/tools/ic50-calculator, accessed on 24 November 2023).

### 4.3. Protein Extraction and Western Blotting Analysis

The cells were grown in 100 mm dishes at 70–80% confluence and were treated with media containing ALA. After 48 h of treatment, the cell monolayers were washed with cold PBS and were solubilized in a RIPA buffer (Cell Signaling Technology, Danvers, MA, USA), plus phenylmethanesulfonyl fluoride (PMSF) at 1 mM concentration. The cell lysates were quantified spectrophotometrically using the Bio-Rad Bradford Assay (Bio-Rad Laboratories, Hercules, CA, USA). All of the samples were loaded on a 10% or 15% SDS–polyacrylamide gel, transferred to a nitrocellulose membrane, and probed with antibodies directed against cyclin D1 (CD1) (ab40754, Abcam, Cambridge, UK), Vimentin (sc-6260), TGFβ-1 (sc-146), GSTP-1 (sc-376481), SOD-1 (sc-8637), Beclin-1 (sc-48341), MAP-LC3β(sc-28266), p62/SQSTM1 (sc-28359), p-mTOR^Ser 2448^ (sc-101738), and Total mTOR (sc-136269) (Santa Cruz Biotechnology, Dallas, TX, USA). As the internal control, all the membranes were probed with anty-glyceraldehyde-3-phosphate dehydrogenase, GAPDH (sc-47724), and Lamin-B (sc-6217) antibodies (Santa Cruz Biotechnology). The antigen–antibody complex was detected through incubation of the membranes for 1 h at room temperature with peroxidase-coupled goat antimouse, antirabbit, or antigoat IgG and revealed using the enhanced chemiluminescence system (Clarity Western ECL Substrate, Biorad, Hercules, CA, USA). The blots were then exposed to film (Santa Cruz Biotechnology). The intensity of bands representing the relevant proteins was measured using ImageJ densitometry scanning software. Nuclear and cytosolic extracts were obtained from cultured cells as described previously [54]. Proteins of the nuclear and cytosolic fractions were determined using a Bio-Rad Bradford Assay (Bio-Rad Laboratories). Equal amounts of proteins (25 μg) were resolved using 10% SDS–polyacrylamide gel electrophoresis, transferred to nitrocellulose membranes, and probed against antibodies to NF-/B (p65) (sc-8008), Nrf2 (sc-365949) (Santa Cruz Biotechnology, Santa Cruz, CA, USA).

### 4.4. Wound-Healing Assay

DU-145 and LNCaP cells were seeded in 24-well plates and grown in complete growth media until a confluent monolayer was formed. At least 12 h before treatment, the complete growth media was replaced with SFM for starvation. The confluent cell sheet was wounded by scratching the culture well surface with a 100 μL pipette tip and cells were washed with 1× PBS to remove any cell fragments or detached cells before incubation in media containing 271 µM and 278 µM of ALA for LNCaP and DU-145, respectively, or DMSO in the controls. Cell migration was monitored by using a Leica DMi8 inverted microscope (Leica THUNDER Imager Live Cell, Wetzlar, Germany) equipped with an environmental chamber in order to maintain cells at 37 °C in a humidified 5% CO_2_ atmosphere. Time-lapse images were captured every 20 min for 48 or 60 h, using an x10 objective and a phase contrast filter. The ImageJ software (version 2.1.0/1.53c) was used to analyze the time-lapse images using the Wound-Healing Size Tool plugin with the following parameters: variance window radius 20, threshold value 20, percentage of saturated pixels 0.001 and variance window radius 20, threshold value 30, and percentage of saturated pixels 0.001 for DU-145 and LNCaP cells, respectively. The following statistics and figures were obtained using R (R version 4.2.1, RStudio version 2021.09.0).

### 4.5. Lipocell Assay

This method is based on the ability of peroxides to promote the oxidation of Fe^2+^ to Fe^3+^. Fe product binds to thiocyanate developing a colored complex with measurable with photometer Free Carpe Diem (Diacron s.r.l., Grosseto, Italy). The absorbance increase was directly proportional to the concentration of the peroxides present in the sample. Then, the cells were sonicated, and the absorbance was read at 505 nm according to the manufacturer’s report. The number of hydroperoxides was calculated according to the following formulas: Abs Sample/Abs Standard ^10^ = nanomoles of hydroperoxides contained in the sample nanomoles of hydroperoxides contained in the sample/number of cells used ^2^ = nanomoles of hydroperoxides million cells.

### 4.6. Immunofluorescence Assay

Prostate cancer cells were seeded into a six-well multiwell and synchronized in SFM for 12 h. Cells were exposed to ALA (271 μM for LNCaP; 278 μM for DU-145) for 24 h and then fixed with methanol followed by 5% bovine serum albumin blocking and incubated with a rabbit antihuman fibronectin antibody (sc-9068), Santa Cruz Biotechnology, Santa Cruz, CA, USA. LNCaP and DU-145 cells were exposed or not to the following treatment: ALA, CQ, and ALA + CQ for 48 h, and incubated with a rabbit anti-MAP-LC3β (sc-28266) and mouse anti p62/SQSTM1 (sc-28359) antibodies overnight. The cells were subsequently incubated for one hour with the following secondary antibodies; anti rabbit donkey alexa fluor 488 and anti mouse donkey alexa fluor 594. For detection of nuclei, staining with 4′,6-Diamidino-2-phenylindole (DAPI, Sigma Aldrich, Milan, Italy) was used. Fluorescence was evaluated by three independent observers using an Olympus BX50 microscope, and the images were taken with CSV1.14 software, using a Leica camera for image acquisition.

### 4.7. Soft Agar Assay

Cells 2 × 10^4^ were plated in 2 mL of 0.35% agarose with 5% FBS in phenol red-free media, in a 0.7% agarose base in 12-well plates. Two days after plating, media containing control vehicle or treatments were added to the top layer, and the media were replaced every 2 days. After 15 days, the images of colony formation were acquired using a Leica DMi8 inverted microscope (Leica THUNDER Imager Live Cell). The ImageJ software (version 2.1.0/1.53c) was used to analyze the images.

### 4.8. Statistical Analysis

Optical densities were measured using the Image J software and their results are presented as fold induction with respect to control. All results are presented as mean ± SD of data from three independent experiments. Data were analyzed by a two-paired Student’s *t*-test by JASP 0.17.2.1 (Intel) (University of Amsterdam, The Netherlands). *p* < 0.05 was considered as statistically significant and the figures were obtained using R (R version 4.2.1, RStudio version 2021.09.0).

## Data Availability

The data presented in this study are available on request from the corresponding author.

## Supplementary Materials

The following supporting information can be downloaded at: https://www.mdpi.com/article/10.3390/ijms242317111/s1.
